# Immunohistochemical study of type III collagen expression during pre and post-natal rat skin morphogenesis

**Published:** 2014-03

**Authors:** Elham Mohammadzadeh, Mohammad Reza Nikravesh, Mehdi Jalali, Alireza Fazel, Vahid Ebrahimi, Ali Reza Ebrahimzadeh-bideskan

**Affiliations:** 1 Department of Anatomy and Cell Biology, School of Medicine, Mashhad University of Medical Sciences, Mashhad, Iran

**Keywords:** Immunohistochemistry, Morphogenesis, Skin, Type III collagen

## Abstract

***Objective(s):*** Skin extracellular matrix, which contains type I and type III collagens, is involved in skin development. The aim of this study was to investigate type III collagen distribution pattern as well as its changes during pre and post-natal skin morphogenesis in rats.

***Materials and Methods:*** Ventral skins of Wistar rat embryos at different stages from 10 to 20 gestational day (E_10_-E_20_) and also one month and one year post natal rat pups were fixed in normalin, embedded in paraffin and 5 µm thick sections were incubated with Anti type III collagen antibody. In order to detect staining intensity, the reactions were observed and graded by three examiners separately. Kruskal-Wallis non-parametric statistical test and SPSS software version 11.5 were used to compare differences between samples.

***Results:*** Immunoreactivity of type III collagen was distributed weakly in the mesenchymal connective tissue on day 10 (E_10_). The observed reaction was increased onE_12_ and E_14_. This reaction was clear in basement membrane, relatively intensive in dermal papillae and moderate in dermal reticularis on E_14_. This immunoreactivity pattern was increased afterward on E_16_, not changed on E_18_ and decreased in dermal reticularis on E_20_. The density of collagen type III in dermal papillae and dermal reticularis in skin of one year old rats were decreased comparing to one month old rats.

***Conclusion:*** Our results showed that type III collagen is expressed and timely regulated during pre and post natal rat skin morphogenesis.

## Introduction

Collagen, an important post-translationally modified protein, in cell-cell interactions during embryonic development, plays a critical role in morphogenesis. Collagen is a family of proteins in all vertebrates which varies in size, function and tissue distribution (1). All members of the collagen family share a characteristic of triple-helical structure composed of three α-chains (2). Of the 28 different types of collagen described to date, type I is the most abundant collagen in the human body and is the most studied type which comprises 90% of bone mass and is the main component of human skin (80%) (3,4). Also type III collagen has a similar tissue distribution as type I collagen, but the ratio of type III to type I collagen varies from tissue to tissue (5, 6). Collagen type III makes up the remaining of skin collagen (15%) (4). Furthermore, collagen type III may share some of the regulatory mechanisms of type I collagen. In addition, the ratio of these two collagens often changes in various tissues during the development (5). In contrast, in rat newborns, collagen type III and I comprises 70% and 30% of the skin, respectively (7).

Moreover, collagens as the most predominant matrix components regarding tissue distribution and quantity are involved in the developmental processes such as angiogenesis, intramembranous ossification as well as tooth germ development (7, 8). It has been shown that collagen type III has a great role in cell attachment and promotion of growth, differentiation and embryo-genesis (7). Several factors, such as autoimmune diseases, aging, and stress can change the quantity and integrity of skin collagen. Skin aging is a complex process that has been created with intrinsic and extrinsic factors. Intrinsic or chronological factor is occurred during life and is affected by genetic factors. These factors cause dermal matrix alterations (8) and impair collagen synthesis as well as degradation and consequently affect skin function. Also, during the aging, the thickness of epidermis decreases because of the decrease in number of keratinocytes (9). Additionally, collagen as an extracellular matrix component was influenced by aging (10). Recent studies have shown that skin collagen content decreases with aging (11).

Little information is available about the distribution and function of type III collagen during embryonic skin morphogenesis and its content changes in skin after birth and aging. Several studies have been done about of collagen amount and changes during aging; we nevertheless do not have exact information about changes of type III collagen during aging. So, here we investigated the distribution of collagen type III during embryonic skin morphogenesis and after birth.

## Materials and Methods

This research was done in Mashhad University of Medical Sciences according to ethics committee guide lines and all protocols of animal experiments have been approved by the Institution's Animal Care Committee.

Thirty virgin Wistar rats (10-week-old and 180–200 g weight) were purchased from Laboratory Animal house of Mashhad University of Medical Sciences, Mashhad, Iran. The animals were maintained in animal house under controlled condition (12 hr light/dark cycle, 21°C and 50% relative humidity) with laboratory chow and water provided *ad libitum*. 

Female rats mated overnight with fertile males of the same strain at a ratio of 3:1. The day on which spermatozoa were found in a vaginal smear was considered to be the zero day of gestation (E_0_). The gestational period of this strain of rats is approximately 21 days. At various gestational days from E_10_ to E_20_, pregnant rats (three rats at each stage) were anesthetized with ether inhalation and fetuses were removed and sacrificed. The skin of the fetuses and the ventral skin of one month and one year old rats were taken immediately, washed in normal saline and fixed in 4% formaldehyde in 0.01 M phosphate buffer (PBS, pH 7.4) overnight at room temperature. After fixation, the tissue specimen were dehydrated with increasing concentrations of alcohol, cleared in xylene and then embedded in paraffin. The paraffin blocks were cut using microtome (MT-XL; RMC, Tucson, AZ, USA) into 5µm thick sections and put on poly L- lysine coated slides (12).

For immunohistochemical staining, the sections were deparaffinized with xylene, rehydrated by increasing concentrations of alcohol and rinsed for 10 min in 0.1 M PBS. After that, the sections were treated with 0.02% hyaluronidase in PBS for 20 min at 37°C for antigen retrieval and immersed in 0.3% methanol containing 1% hydrogen peroxide for 30 min to block endogenous peroxidase and rinsed in 0.05 M PBS plus 0.025% Trition X-100 for 10 min at room temperature. Then, the sections were incubated in 10% normal goat serum with 1% BSA in PBS for 30 min at room temperature. Subsequently, all sections were covered with the primary antibody (Rabbit IgG polyclonal to collagen III, diluted 1/400 in PBS with 1% BSA) and incubated overnight at 4°C. After washing the sections in PBS containing 0.025% Trition X-100 for 10 min, the HRP- conjugated secondary antibody (goat anti rabbit IgG) was diluted at the ratio of 1:1000 in PBS with 1% BSA and applied to sections for 2 hr at room temperature. The antigenic sites were demonstrated via reaction of sections with a mixture of 0.03% DAB in 0.05 M PBS buffer, pH 7.6, containing 0.01% H_2_O_2_ for 10 min. The sections were counterstained with Mayer’s hematoxylin and finally were dehydrated in increasing concentrations of alcohol, cleared in xylene and mounted.

In order to detect staining intensity, the reactions of the tested antibody (five slides for each stage) were observed by three examiners blindly with Olympus microscope (AH-2, Japan). On the basis of their staining intensity, the sections were graded as very weak (+), weak (++), moderate (+++) and strong (++++) (13).

The immunoreactive specificity was checked by negative and positive controls as follows:

1. Negative control: a control incubated with normal mouse serum instead of the primary antibody
2. Positive control: adult rat skin with the corresponding antigen was incubated with antibodies overnight at 4°C.

***Statistical analysis***

SPSS software version 11.5 and Kroskal-Wallis non-parametric statistical test were used to compare differences between samples taken from different gestational days, one month and one year old rat offspring. Significance was determined at *P*≤0.05 whereas non significance is indicated (NS).

## Results

**Figure1:**
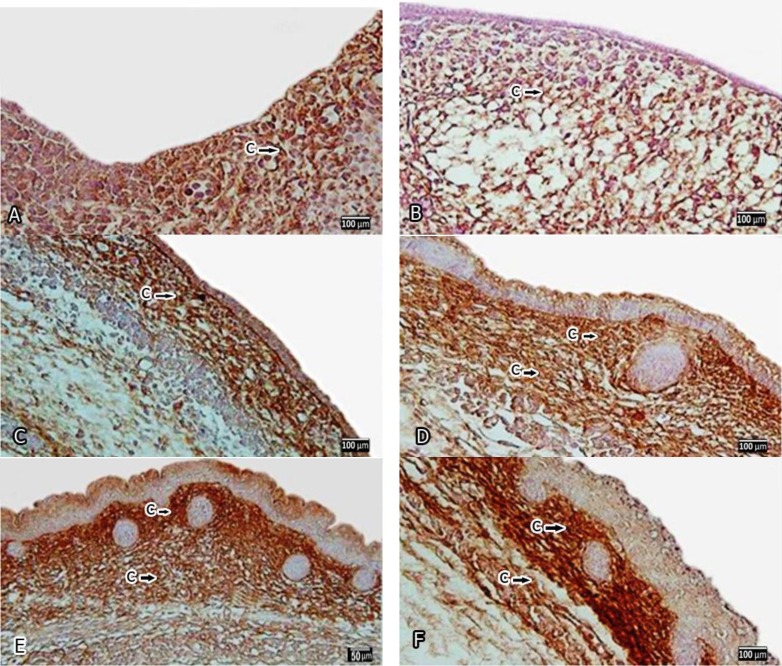
Photomicrographs that show the immunolocalization of type III collagen of rat developing skin A, B, C, D, E and F are representative of days 10, 12, 14, 16, 18 and 20 of gestation, respectively. Immunoreactivity is shown with thin arrow in corium in A, B , and C and dermal papillae in D, E, and F. Thick arrow points to immunoreactivity in dermal reticularis, collagen(C)

Immunohistochemical staining showed changes of type III collagen distribution in the developing skin on various gestational days from E_10_ to E_20_. In fetuses on E_10_, immunoreactivity specific for type III collagen was weakly and sparsely distributed in the mesenchymal connective tissue (1.125±0.6292) and it was relatively distinct on a fairly wide region of mesenchymal connective tissue just beneath the epithelium of the developing skin, where will be formed dermal papillae (Figure 1A). The observed reaction for type III collagen, which had been seen in the mesenchymal tissue behind the epithelium, was slightly stronger in fetuses on E_12 _comparing to E_10_ (1.25±0.5000) but it was not significant. At this embryonic stage, the embryos were covered with an epithelium composed of one layer of cuboidal cells (ectoderm) which reacted with hematoxilin and could be clearly distinguished but showed no immunoreactivity (Figure 1B).

In fetuses, on E_14 _specific immunoreactivity for type III collagen was differentially distributed in different regions of the developing skin, so it was more distinct than previously stage (E_12_). The reaction of the mesenchymal connective tissue just beneath the epithelium, Corium, which will form the dermal papillae, was observed (2.75±0.5000) but the intensity of the reaction was not significant comparing to pervious stage. At this embryonic stage, the above mentioned immunoreactivity was appeared on the developing basement membrane region. At the same time, specific immunoreactivity for type III collagen appeared very slightly and weakly in the mesenchymal tissue under the corium, which is called subcorium that will be developed to hypodermis. At this stage, total thickness of the epithelium was increased and developed into two layers including a cuboidal basal cell layer and a flattened superficial cell layer, known as epitrichium or periderm. Each epithelial cell still had a large round nucleus. On their free surface side above these epithelial cells, peridermal cells, in a single layer, became to some extent swollen and elliptical (Figure 1C). Examination of specimens from fetuses on E_16_ showed that the immunoreactivity pattern was increased at this stage, but changes of immunoreactivity was not significant compared to E_14_, dermal papillae was distinguished from dermal reticularis and the density of type III collagen in dermal papillae (3±0.58165) was more distinct than dermal reticularis (1.75±0.5000). Also, epithelium had gradually become stratified that the cells nuclei were stained deeply with hematoxilin (Figure 1D).

In fetuses, on E_18 _immunoreactivity was restricted to a very narrow region of connective tissue, dermal papillae (3.25±0.5000). However, this reaction was not so distinct in the region under the dermal papillae, known as dermal reticularis (2.25±0.5000) and variation in intensity of reaction between this stage, E_10_ and E_12_ was significantly (*P*<0.05). The surface layer of squamous stratified epithelium, which was not stained deeply by hematoxilin, appeared to be parakeratin-ized, but the nuclei were still visible in the surface layer of epithelial cells (Figure 1E).

**Figure2:**
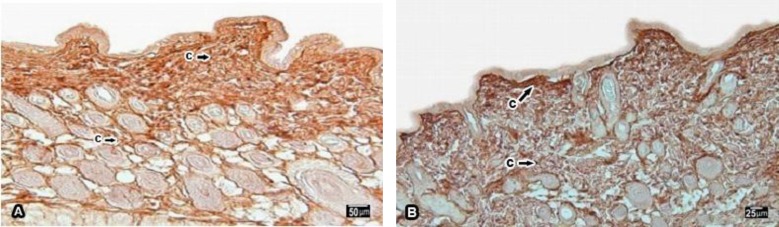
Photomicrographs that show the immunolocalization of type III collagen in the skin of one month post natal rat offspring, immunoreactivity (thin arrow) in dermal papillae, Collagen(C), immunoreactivity in dermal reticularis (thick arrow)

On embryonic day 20 (E_20_), immunoreactivity specific for type III collagen was observed like to E_18_ in dermal papillae (3±0.8165) and there was not any difference in staining intensity comparing to the pervious embryonic day (E_18_) but the intensity of immunoreactivity in dermal reticularis was decreased compared to the previous stages (0.875±0.2500) and the immunoreactivity varied significantly between this stage and E18 (P<0.05). In addition, intensity of this reaction was detected mainly around the developing hair follicles located in dermal reticularis. 

At this embryonic stage, the basal and intermediate cell layers in epidermis had a large nucleus. The layer of epithelial cells that were the closest to the surface was significantly flattened, and periderm cells, which had been to some extent swollen and elliptical on E_18_, had disappeared completely.

The keratinized surface layer was not stained with hematoxiline, and the nuclei were absent from cells in the keratinized layer of epiderm. Each interpapillary cell column was still restricted to a very narrow region, and the surface layer of this region, which was not stained deeply by hematoxiline, appeared to be parakeratinized, and the nuclei were still visible in cells of the surface layer of the interpapillary epidermal cell columns (Figure 1F).

The result of 1 month post natal rat offspring showed that collagen type III was existed in both of dermal papillae (3.5±0.5774) and reticular dermis (2.25±0.5000) but immunoreactivity was detected to be lower in dermal reticularis (Fig. 2A). Additionally, the amount of collagen type III in one year old rats was decreased in both of dermal papillae (3.25±0.5000) and dermal reticularis (1.75±0.5000) rather than 1 month rat skin but changes of immunoreactivity was not significant(Figure 2B).

## Discussion

Our results suggested that type III of collagen existed in extra cellular matrix in primary stage of development and during the skin development, the amount of this type of collagen gradually increased in skin. During rat skin development, two parts was formed: 1- dermal papillae and 2- dermal reticularis that expression of collagen type III, but this type of collagen in dermal papillae was more than dermal reticularis. Additionally, type III of collagen plays important role in basal membrane development. In one study, this type of collagen was observed close to basal lamina, skin appendix, blood vessels endothelium, nerve and muscular fibers (14). Also collagen type III existed in reticular lamina in basal lamina as a part of reticular fibers (15). In the latest stage of development, expression of collagen type III decreased in dermal reticularis but in dermal papillae its expression was ascending until birth. After birth, the expression of collagen type III in dermal papillae and dermal reticularis was seen but during the aging, the amount of it decreased. Regarding the human embryo, it was shown that collagen type I and III, were distributed equally across the dermis and connective tissues under dermis with an aggregation in dermal-epidermal junction (DEJ). In the other research that was done on chick embryo, collagen type III has not been found from E_6_ to E_9 _(15). In parallel, in a study on human embryo, it was shown that dermis was organized by individual and fine collagen fibrils covering the surfaces of mesenchymal cells that with aging were increased abundantly in extra cellular matrix. Diameter of fibrils was increased and in the 15^th^ week of pregnancy the dermis was divided into dermal papillae and reticularis (16). Our results have shown that collagen expression increased during skin development but the rate of collagen expression decreased near the birth. In addition, in the skin of chicken embryo, synthesis of collagen type III reached its maximum from E_8 _to E_12_. Moreover, in other studies results were shown that collagen type III expression was decreased before the birth but after the birth its expression has been continued at a lower level compared to collagen type I. Shuttleworth and Forrest represented that collagen type III is essential skin collagen for normal dermal development in embryo that is necessary for normal ectodermal development (17). Also, our results confirm that collagen type III existed from primary developmental stage and its quantity increased during development but suddenly decreased near the birth.

On the other hand, our result showed abundant expression of collagen type III in dermal papillary in one month old rats but this fibrils were less in dermal reticularis compared to dermal papillae. collagen type III were decreased during aging as this fibril's density were decreased in dermis. In addition, other studies have shown that during the aging, structurally epidermis probably becomes thinner, adherence of corneocytes was declined and dermoepidermal interface was flattening. Also, it was observed that the number of melanocytes and Langerhans cells was decreased and the dermis became atrophic which was comparatively acellular and avascular. Furthermore, dermal collagen, elastin and glycosaminoglycans were altered resulting in wrinkles, laxity and fragility of aged skin (8, 18, 19).

## Conclusion

Our results showed that type III collagen is expressed and timely regulated during pre and post natal rat skin morphogenesis. Also, at the latest stage of development, expression of collagen type III decreased in dermal reticularis but in dermal papillae its expression was ascending until birth. After birth, the expression of collagen type III in dermal papillae and dermal reticularis was seen but during the aging, its amount decreased.
